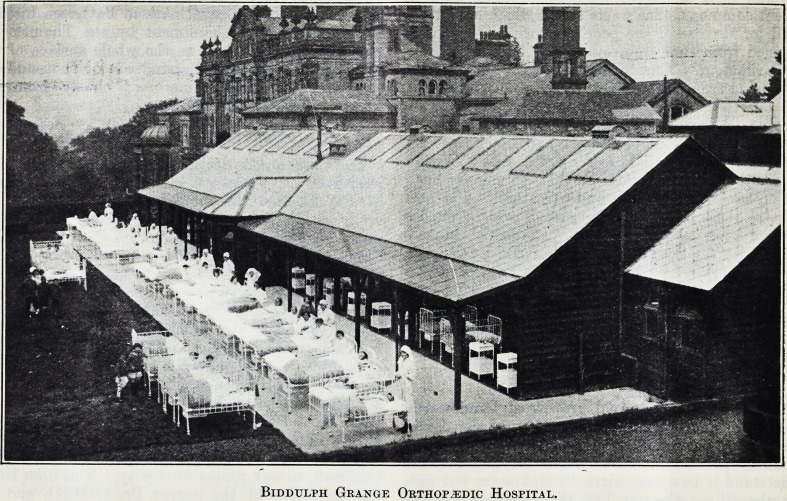# Cripples at Biddulph Grange

**Published:** 1924-12

**Authors:** 


Biddulph Grange Orthopædic Hospital.

### CRIPPLES AT BIDDULPH GRANGE.

The new orthopædic hospital at Biddulph Grange, Staffordshire, the opening of which by the Prince of Wales was recorded in our issue of last July, is now in full working order. During the last twelve months most of the fine rooms have been completely transformed and many structural alterations made. These include spacious open-air wards, as shown in our illustration, to which the beds of the patients are wheeled and where, shielded from cold winds, a maximum amount of sunshine can be obtained. The gardens of the new hospital are particularly beautiful, possessing a unique feature in the Chinese garden, planned to represent the famous willow pattern. Inside the building everything possible has been done to make the hospital up-to-date and thoroughly efficient, while the accommodation is sufficient to cope with the whole of the cripple cases in the county. The expensive task of equipping a modern orthopædic hospital has been carried out by Mr. Ronald Copeland, assisted by three other gentlemen, but money is still needed if the institution is to continue its beneficent work unfettered.

## Figures and Tables

**Figure f1:**